# ESRRG downregulation in early spontaneous abortion induces mitochondrial damage, leading to impaired trophoblast function

**DOI:** 10.1080/07853890.2026.2622749

**Published:** 2026-02-02

**Authors:** Sha Lv, Lieyang Li, Xiaoxiao Xu, Zhengwei Liang, Rongrui Zhang, Zunlun Zhou, Deqin Lu

**Affiliations:** ^a^ Department of Gynaecology, Affiliated Hospital of Guizhou Medical University, Guiyang, China; ^b^ Department of Obstetrics, Affiliated Hospital of Guizhou Medical University, Guiyang, China; ^c^ Department of Pathophysiology, Guizhou Medical University, Guiyang, China; ^d^ Guizhou Provincial Key Laboratory of Pathogenesis and Prevention of Common Chronic Diseases, Guizhou Medical University, Guiyang, China

**Keywords:** Early spontaneous abortion, ESRRG, trophoblast dysfunction, mitochondria

## Abstract

**Background:**

Early spontaneous abortion (ESA) is recognized as the most common complication during pregnancy and is often linked to dysfunction in the trophoblast and placenta. Studies suggest that downregulation of trophoblast oestrogen-related receptor gamma (ESRRG) may play a significant role in the impairment of placental function. In light of these findings, we evaluated the impact of ESRRG on trophoblast and placental function in ESAs, aiming to uncover new targets for diagnosis and treatment.

**Patients/materials and methods:**

Bioinformatics methods were used to analyse differentially expressed genes in the trophoblast of ESA patients and normal controls. ESA Patients and controls were recruited and the villus tissues were collected. Protein and mRNA levels were determined by western blot and qRT–PCR, respectively. Mitochondrial morphological changes in trophoblasts were observed *via* transmission electron microscopy. CCK8 and Transwell assays were conducted with ESRRG-knockdown HTR-8/SVneo cells. MitoSOX staining, JC-1 assays and ATP quantification were used to assess mitochondrial function *in vitro*. In addition, Esrrg was overexpressed in ICR female mice, and the number of embryos in the uterus was determined.

**Results:**

The expression of ESRRG was significantly decreased in the placental villous tissue of ESA patients, accompanied by abnormal mitochondrial morphology and decreased ATP levels in trophoblast cells. Impaired proliferation, invasion, migration and tube formation abilities were observed in ESRRG-downregulated HTR-8/SVneo cells, as well as impaired mitochondrial function. ESRRG overexpression was associated with improved trophoblast functionality in a lipopolysaccharide-induced abortion model in ICR mice, leading to an increased number of retained embryos in the uterus.

**Conclusion:**

In summary, this study revealed that ESRRG downregulation plays an important role in ESA, providing new targets for diagnosis and treatment.

## Introduction

Early spontaneous abortion (ESA), or early pregnancy loss, occurs within the first 12 weeks of gestation and is the most common complication of pregnancy [1,2]. The aetiology of ESAs is complex and involves chromosomal abnormalities, endocrine disorders, immune system dysregulation and infections, etc [1,3], but many of the underlying mechanisms remain obscure. ESA causes considerable harm to a woman’s physical and mental health as well as to their families, underscoring the clinical significance of further exploration of its underlying mechanisms. In the initial stages of a normal pregnancy, trophoblast cells exhibit behaviours akin to those of tumour cells, including proliferation, differentiation, invasion, and migration into the uterine decidua and myometrium. They also secrete vasoactive factors to promote angiogenesis and the establishment of the maternal-fetal vascular interface. These processes are crucial for the implantation of the embryo, the development of the placenta, and the maintenance of the pregnancy [4]. A significant amount of research suggests that the proliferation and invasion of trophoblast cells are impaired in cases of spontaneous abortion, highlighting their vital importance in associated pathophysiological mechanisms [5–7]. Research has demonstrated that oestrogen-related receptor gamma (ESRRG) plays a critical role in regulating the proliferation, invasion, and migration of the trophoblast cell line HTR8/SVneo [8,9]. Our previous study indicated a possible association between placental dysfunction in preeclampsia and the downregulation of ESRRG [10]. We speculated that ESRRG might participate in the occurrence and development of ESAs by regulating the biological behaviour of trophoblast cells, thereby affecting the development and function of the placenta. However, there are currently no relevant reports on the role of ESRRG in ESAs.

ESRRG is a member of the oestrogen-related receptor (ERR) family, which has structural similarities to oestrogen receptors; however, it generally does not bind to oestrogen and functions independently of the oestradiol-oestrogen receptor signalling pathway. As a transcription factor, ESRRG is integral to nuclear signalling pathways and the regulation of gene transcription [11,12]. ESRRGs play a significant role in various biological processes, including energy metabolism and embryonic development. It promotes the differentiation of embryonic cells and the formation of tissues and organs by regulating the expression of related genes [13]. Some trans fatty acids and unsaturated fatty acids function as endogenous ligands for ESRRG; upon binding, these ligands can modulate its transcriptional activity, thereby regulating the expression of genes involved in energy metabolism and affecting the cellular uptake and utilization of energy substrates [14,15]. Prostaglandins are integral to the regulation of various physiological processes such as cell proliferation, differentiation, and inflammatory responses. This regulatory function is mediated through their interaction with and activation of ESRRG, a critical factor for sustaining normal tissue and organ functionality [16]. Research has demonstrated a positive correlation between the expression of ESRRG and renal function. ESRRG acts as a transcription factor that directly influences the expression of genes related to renal mitochondrial function, thereby playing a critical role in sustaining normal mitochondrial activity within the kidneys [17]. Mitochondria are the primary sites for biological oxidation and energy conversion, and they are vital for various biological processes, including cellular growth, division, migration, and invasion [18]. The importance of mitochondrial function is particularly pronounced in trophoblast cells, which have increased energy demands [19]. During the early stages of pregnancy, trophoblast cells require significant energy to facilitate rapid proliferation, differentiation, maintenance of invasive capabilities, and the synthesis and secretion of hormones crucial for pregnancy. In the intermediate and later stages of gestation, these cells continue to require substantial energy to support their highly active metabolic functions, which are essential for foetal growth and development [20]. Increasing evidence suggests that mitochondrial dysfunction may be linked to adverse pregnancy outcomes, including miscarriage, preeclampsia, and gestational diabetes [21]. Studies indicate that ESRRG is integral to the regulation of mitochondrial function in chorionic trophoblast cells and that reduced expression of ESRRG leads to a decrease in the expression of specific genes associated with mitochondrial biogenesis and energy metabolism [22,23].

Our study analysed a dataset from the Gene Expression Omnibus (GEO), which contains RNA-sequencing data of placental tissue from ESA patients and normal controls. The results indicated a significant decrease in the expression of ESRRG in ESA patients. We speculated that the downregulation of ESRRG led to impaired trophoblast function, thereby contributing to the occurrence of early miscarriage, and that the underlying mechanism may be related to the induction of mitochondrial damage. Therefore, we compared the expression levels of ESRRG in chorionic tissues from patients with normal pregnancies with those from patients who experienced early spontaneous abortions. The effects of ESRRG on the proliferation, invasion, and migration capabilities of trophoblasts were subsequently assessed *in vitro* and *in vivo*. Our findings provide novel insights into the pathogenesis of ESA and identify potential targets for its diagnosis and treatment.

## Patients/materials and methods

### Identification of genes related to ESAs

The RNA-seq dataset GSE123719 (this study has not yet been published) was analysed using GEO2R and downloaded from the GEO database. The significant differentially expressed genes (DEGs) were identified based on the following criteria: *adj. p* < 0.05, |log_2_FC| > 2, and baseline > 1000. The results were visualized *via* volcano plots. The ChIP-seq dataset GSE104905 [17] was downloaded from the GEO database to screen for possible genes regulated by ESRRG. The RNA-seq dataset GSE28551 [24] was analysed to assess whether there is a difference in the expression of ESRRG in the human placenta between the third trimester (from uncomplicated pregnancies that were delivered at term) and the first trimester (from healthy women undergoing surgical abortion at 9–12 weeks). The RNA-seq dataset GSE9984 [25] was used to analyse the expression of ESRRG in the human placenta of the first trimester (45–59 days), the second trimester (109–115 days) (from uncomplicated elective termination) and the term (from caesarean section).

### Gene functional enrichment analysis

Gene set enrichment analysis (GSEA) [26] was performed to explore the biological pathways associated with the DEGs. An enrichment analysis of these downregulated DEGs in relation to Gene Ontology (GO) and Kyoto Encyclopedia of Genes and Genomes (KEGG) [27] signalling pathways was subsequently performed *via* R4.4.2. The GO enrichment analysis contains three categories: biological process (BP), cellular component (CC), and molecular function (MF). An *adj. p* value < 0.05 was considered statistically significant.

### Human tissues

All experiments involving human subjects were approved by the Ethics Committee of the Affiliated Hospital of Guizhou Medical University (Approval Number: 2023 Lunshen No. 906), and written informed consent was obtained from each donor. The study adheres to the Declaration of Helsinki. ESA patients (*n* = 20) and pregnant women who voluntarily requested induced abortion for the personal reasons as controls (*n* = 20) were included. For ESA patients, the absence of foetal cardiac activity as confirmed by ultrasound was used as the diagnostic criterion, excluding individuals with endocrine and metabolic disorders, autoimmune diseases, anatomical abnormalities of the reproductive tract, or a history of medication use during pregnancy. The relevant information and parameters of the participants are presented in Table 1. Fresh placental villous samples were thoroughly rinsed with isotonic sodium chloride solution prior to experimentation.

**Table 1.** Characteristics of the enrolled participants.

Characteristics	Control (*n* = 20)	ESA (*n* = 20)	*p* value
Age, mean [median; range], years	29 [29; 23–37]	31.75 [30; 22–40]	NS
Days of gestation, mean [95% CI of mean], days	55.9 [51.68–60.12]	59.1 [57.54–60.66]	NS
Gravidity, mean [95% CI of mean]	2.3 [1.77–2.83]	2.1 [1.44–2.76]	NS
Previous abortions, mean [95% CI of mean]	0.45 [0.13–0.77]	0.5 [0.01–0.99]	NS

### Immunohistochemistry (IHC) and immunofluorescence (IF)

Placental villi tissues were collected, fixed with 4% paraformaldehyde, embedded in paraffin according to conventional procedures, and sectioned into 5-μm-thick slices. The sections were sequentially dewaxed, hydrated, and subjected to antigen retrieval. The samples were then incubated with H_2_O_2_ to block endogenous peroxidase activity. After the samples were blocked with goat serum, primary antibodies (Table S1) were applied to the sections, which were then incubated at 4 °C overnight. After the samples were washed with PBS, the secondary antibodies for IHC or IF were separately incubated at room temperature for 45 min. The sections prepared for IHC were stained with 3,3′-diaminobenzidine (DAB) and counterstained with haematoxylin. After dehydration, clearing and mounting, the sections were observed under an upright microscope (BX53, Olympus, Japan). The sections prepared for IF were incubated with DAPI at 37 °C for 10 min in a light-protected environment and mounted with an antifluorescent quenching agent. Observations and image acquisition were conducted using an inverted laser scanning confocal microscope (IXplore SpinSR, Olympus, Japan).

### Quantitative real-time PCR

Human chorionic tissue and total RNA were extracted using a Quick Extraction Kit for Micro Total RNA (TR150, Genstone Biotech, Beijing, China), and the concentration of the RNA was quantified using a Nanodrop spectrophotometer (Thermo). A total of 500 ng of RNA was reverse transcribed into complementary DNA (cDNA) using a reverse transcription kit (RR047A; Biosharp, Beijing, China). Real-time PCR was carried out with SYBR Green qPCR Master Mix (HY-K0501, MCE, USA) and the Bio-Rad CFX Manager system (Bio-Rad, USA). The primer sequences utilized were as follows: for β-ACTIN, the forward primer (5′–3′) was CTCCATCCTGGCCTCGCTGT, and the reverse primer (5′–3′) was GCTGTCACCTTCACCGTTCC; for ESRRG, the forward primer (5′–3′) was AGCTGGCAAGATGCTGATGA, and the reverse primer (5′–3′) was CAGACCTTGGCCTCCAACAT; for CKMT1A, the forward primer (5′–3′) was CTCACCACACCCTTCACCTC, and the reverse primer (5′–3′) was TGTGTTTGCTGCTCACAGGA; for CKMT1B, the forward primer (5′–3′) was GGCAGAGATTCCCTGACGAC, and the reverse primer (5′–3′) was CACAGGTCTCCAGACACCAC; for Ckmt1, the forward primer (5′–3′) was GATTGACTGTGAACGGCGTC, and the reverse primer (5′–3′) wasGGGTCTTGACTCATCCGCTG. The resulting data were analysed *via* the 2^−ΔΔCt^ method.

### Western blotting

Total protein was extracted from human and mouse tissue samples as well as from cultured cells using RIPA lysis buffer (HY-K1001, MCE, USA). The concentration of the extracted proteins was quantified *via* the bicinchoninic acid (BCA) assay (PC0020, Solarbio, Beijing, China). The prepared protein samples were separated *via* SDS–polyacrylamide gel electrophoresis, followed by transfer to PVDF membranes. The membranes were subsequently blocked with 5% skim milk and incubated overnight with a primary antibody (Table S1). Following this incubation, the membranes were incubated with a secondary antibody, and images were captured *via* a chemiluminescence imaging analysis system.

### Haematoxylin-eosin (HE) staining

Paraffin sections of chorionic tissues were dewaxed and hydrated, stained with haematoxylin for 10 min, rinsed with running water, and then stained with eosin for 3 min. After dehydration, the sections were mounted with neutral resin and examined under an upright microscope, and images were captured for analysis.

### Dihydroethidium (DHE) staining for determination of reactive oxygen species (ROS)

Paraffin sections of chorionic tissues were dewaxed and hydrated, followed by three washes with PBS. A working solution of 20 nM DHE (HY-D0079, MCE, USA) was applied to ensure complete coverage of the tissues. The sections were then incubated at 37 °C and shielded from light for 30 min, followed by staining with DAPI for 10 min. The washed sections were subsequently treated with an antifluorescent quenching agent. The slices were examined using a laser scanning confocal microscope, and images were captured for analysis.

### Transmission electron microscopy (TEM)

Fresh chorionic tissue samples were preserved in 2.5% glutaraldehyde overnight, followed by fixation in 2% osmium tetroxide for 2 h. The samples were washed twice with distilled water, stained with 1% uranyl acetate for 30 min, and subsequently washed twice with PBS prior to dehydration. The samples were then embedded, sectioned, and stained with uranyl acetate and lead citrate. Imaging was conducted *via* TEM (HC-1, Hitachi, Japan).

### Cell culture and ESRRG-knockdown

HTR-8/Svneo cells (ZQ0482, Zhong Qiao Xin Zhou, Shanghai, China) were cultured in 1640 medium (ABW, Shanghai, China) supplemented with 10% foetal bovine serum (FBS) and 1% penicillin–streptomycin and were incubated at 37 °C in a 5% CO_2_ environment. ESRRG siRNA (Gene Pharma, Shanghai, China) was introduced into HTR-8/Svneo cells at a concentration of 60 nM. The cells were collected 24 h after the transfection process, for subsequent experiments. The sequences for siESRRG were as follows: sense: 5′-GCCCAAGAGACUGUGUUUATT-3′, antisense: 5′-UAAACACAGUCUCUUGUGGGCTT-3′; the sequence for the negative control (NC) was as follows: sense: 5′-UUCUCCGAACGUGUCACGUTT-3′, antisense: 5′-ACGUGACACACGUUCGGAGAATT-3′.

### Cell counting kit-8 (CCK8) assay

The cells were transfected with si-ESRRG and inoculated into 96-well plates. After 24 h of incubation at 37 °C, a 10% CCK-8 (CA1210, Solarbio, Beijing, China) solution was added, and the mixture was incubated for an additional 2 h at 37 °C in the dark. The absorbance at 450 nm was measured using an enzyme marker (Thermo Fisher Scientific, Massachusetts, USA) (OD 450 nm).

### Cell invasion assay

Cells were seeded in the upper chamber (8 μm, Corning, NY, USA) of a Transwell that had been precoated with Matrigel (ABW, Xiamen, China). The lower chamber was filled with 600 µL of medium supplemented with 20% FBS. After a 48-h incubation at 37 °C, the cells were fixed with 4% formaldehyde for 30 min and then stained with 0.1% crystal violet for 15 min. Unstained cells were removed using a cotton swab, and images were captured with an inverted microscope.

### Cell migration assay

The cells were cultured in 6-well plates. Upon achieving over 90% confluence, a 1 μL pipette tip was used to generate uniform scratches across the wells. Following the removal of the disrupted cells through rinsing with PBS, the medium was replaced with serum-free medium. Images of the scratches were obtained at 0, 6, 12 and 24 h *via* phase contrast microscopy.

### Mitochondrial ROS (mROS) and mitochondrial membrane potential (MMP) measurements

Levels of mROS were measured using the MitoSOX Red (HY-D1055, MCE, China) mitochondrial superoxide indicator according to the manufacturer’s instructions. HTR-8/SVneo cells were cultured in 24-well plates and transfected with si-ESRRG for 24 h. The cells in all groups were incubated with a 5 μM MitoSOX Red working solution for 10 min at 37 °C in the dark. After washing three times with warm 1640 culture medium, we detected the ROS intensity with a fluorescence microscope (CKX53, Olympus, Japan).

The MMP was measured with a 5,5′,6,6′-tetrachloro-1,1′,3,3′-tetraethylbenzimidazolyl carbocyanine iodide (JC-1) staining kit (C2003S, Beyotime, Shanghai, China) according to the manufacturer’s instructions. The cells were incubated with JC-1 reagent working solution for 20 min at 37 °C in the dark. The stained cells were subsequently analysed *via* fluorescence microscopy.

### Adenosine triphosphate (ATP) concentration

The ATP concentration of tissues and cells was calculated using an ATP assay kit (HY-K0314, MCE, USA) by measuring chemiluminescence with a luminometer plate reader (GLOMAX20/20, Promega Biotech Co. Ltd., Beijing, China).

### Animal experiments

All animal procedures were approved by the Animal Ethics Committee of Guizhou Medical University (Approval No. 2305209). All studies involving experiments on live animals meet the Animals in Research: Reporting *In Vivo* Experiments (ARRIVE) guidelines. Nine-week-old ICR male and female mice were acclimatized for one week at 22 ± 2 °C, maintained on a 12 h light/dark cycle, and provided ad libitum access to food and water in a specific pathogen-free facility. Each individual mouse serves as one experimental unit. These were exploratory experiments and there were no similar study for reference. Based on statistical principles, experimental error control, and the need for data reliability, sample should have at least 3 replicates in each experiment. Since it is necessary to sacrifice female mice for sample collection, every effort was made to minimize the number of sacrifices while ensuring a sufficient sample size for statistical analysis. However, taking into account the possible situations of infertility or death of the mice, the number of mice in each group was finally scheduled to be seven. The female mice were randomly divided into two groups: the Esrrg overexpression (OE-Esrrg) group (*n* = 14) and the vector control group (*n* = 14). A random number generator was used to generate a random number for each mouse. Then the mice were sequentially assigned to OE-Esrrg and control groups, according to the magnitude of the random numbers. The mice in the OE-Esrrg group were injected *via* the tail vein with 200 μl of a lentivirus overexpressing Esrrg (LV-OE-Esrrg) at a titre of 3 × 10^7^ TU (GeneAdv, Jiangsu, China), whereas those in the vector group were injected with 200 μl of an empty-vector lentivirus at the same titre. Two weeks later, male and female mice were caged together at a ratio of 1:2. When a vaginal plug was observed in female mice, it indicated the start of pregnancy (the corresponding date was recorded as gestational day GD 0.5). Each group was given a booster injection of the same type and dose of virus as the previous injection. Both the OE-Esrrg group and the Vector group were further randomly divided into an abortion model subgroup and a control subgroup (*n* = 7/group), respectively. In the abortion model subgroup, a mouse abortion model was established on GD 7.5 by intraperitoneal injection of LPS (LPS was calculated at 250 μg/kg and dissolved in 100 μl of normal saline for injection) [28], whereas the control subgroup was given an intraperitoneal injection of 100 μl of normal saline. Twenty-four hours later (GD 8.5), the mice were sacrificed under pentobarbital sodium anaesthesia. The uteri were dissected out, and embryonic tissues were collected. The number of viable embryos in the uteri of each group was counted. The villous tissues were isolated for western blot analysis. Two female mice which were not pregnant for over 2 weeks were excluded. Since the number of times of treatment is small (only three times for each mouse), the corresponding drug treatments were administered to the mice in each group in a randomized order. Due to the small number of mice and the large size of the room, the environmental differences are negligible. The cages were randomly assigned to positions within a fixed area.

### Statistical analysis

ImageJ and R 4.4.2 were used to analyse the image results and relevant data. The data are expressed as the mean ± standard deviation (SD). Differences between two groups were assessed using a Student’s two-tailed t test. Differences among more than two groups were analysed using a one-way analysis of variance (ANOVA) with graphical representation. Statistical significance was defined as a *p* < 0.05.

## Results

### Significant DEGs and functional enrichment

**Figure 1.**
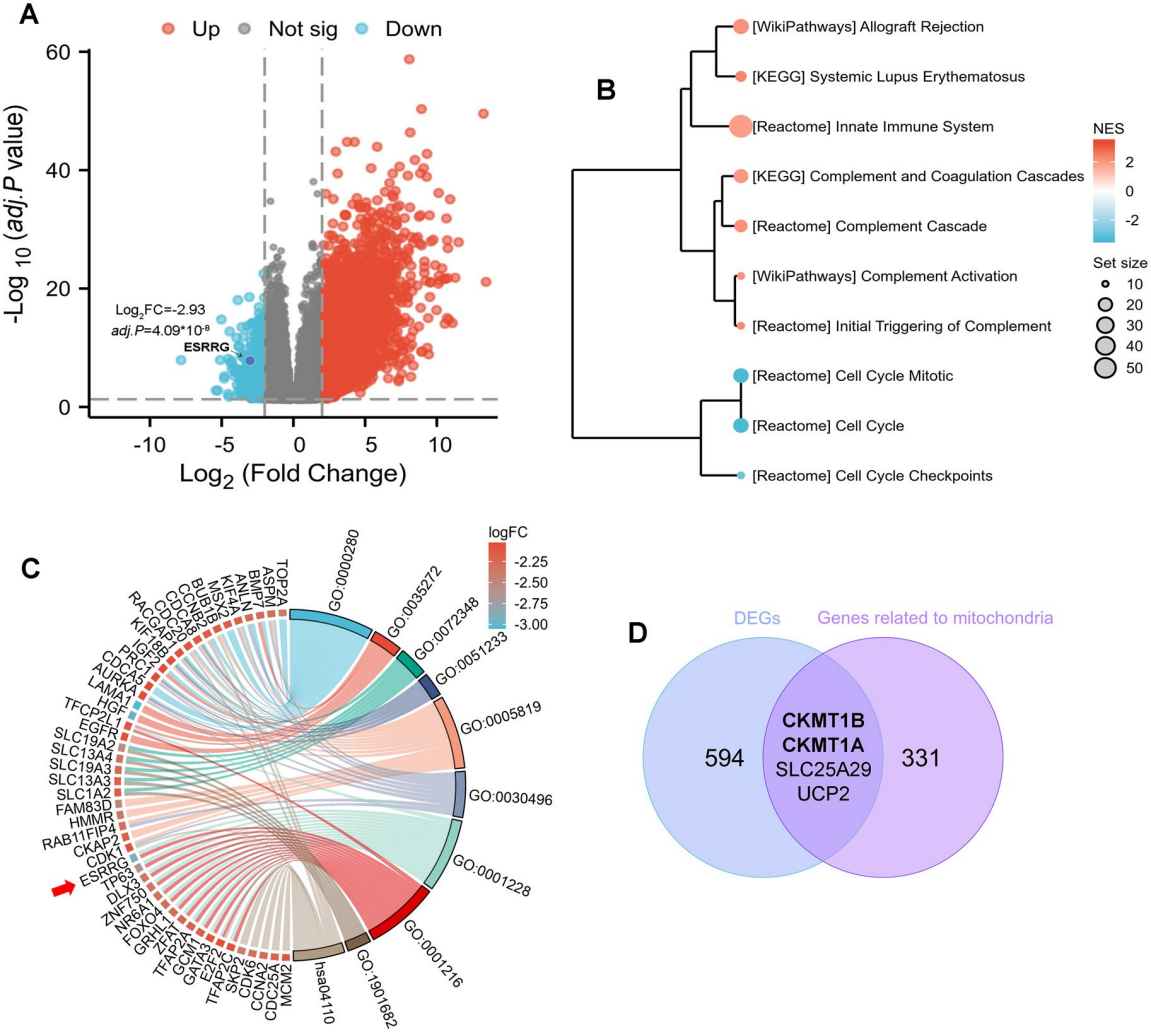
DEGs from the GSE123719 dataset and their functional analyses. (A) Volcano plots showing the DEG distribution. (B) GSEA visualizing the functional enrichment results. (C) Chord diagram illustrating the GO-KEGG enrichment of 184 significantly downregulated DEGs. (D) Venn diagram of the 598 DEGs and 335 genes related to mitochondrial function.

Five hundred and ninety-eight significant DEGs were identified from the GSE123719 dataset, including 414 upregulated and 184 downregulated genes. ESRRG was one of the downregulated genes (Figure 1A). GSEA (Figure 1B) showed that the highly expressed genes were associated primarily with the immune response and allogeneic rejection, which was potentially attributed to dead embryos. The downregulated genes were involved mainly in mitosis and the cell cycle. Considering that gene knockdown may lead to infertility, it is unsuitable as a method for miscarriage treatment or for establishing a pregnant mouse model. We focused on genes that were significantly downregulated in miscarriages. GO-KEGG analyses (Figure 1C and Table 2) revealed that the 184 downregulated DEGs were enriched primarily in DNA-binding transcription activator activity, RNA polymerase II-specific and DNA-binding transcription repressor activity in MF; nuclear division, organelle fission and mitotic cell cycle phase transition in BP; the spindle and midbody in CC; and the cell cycle in KEGG. ESRRG plays a key role in DNA-binding transcription, implying that downregulation of ESRRG may contribute to miscarriage by binding to specific DNA sequences and modulating gene transcriptional activity, thereby influencing cellular physiological functions and developmental processes. We downloaded 335 genes that were identified as being directly related to mitochondrial functions in the KEGG database and performed a cross-analysis with the DEGs, resulting in the downregulated genes creatine kinase, Mitochondrial 1B (CKMT1B) and creatine kinase, Mitochondrial 1 A (CKMT1A) and the upregulated genes solute carrier family 25 member 29 (SLC25A29) and uncoupling protein 2 (UCP2) (Figure 1D), which may participate in the impairment of mitochondrial function in ESAs. The analysis of RNA-seq data from the GSE28551 dataset demonstrated that ESRRG expression in human placental tissue during the third trimester was mildly up-regulated compared to the first trimester (log2FC = 0.56, *p* < 0.05). Similarly, in dataset GSE9984, ESRRG levels were slightly elevated in the second trimester compared to the first trimester (log2FC = 0.16, *p* < 0.05), and more notability increased in term placentas compared to the first trimester (log2FC = 0.58, *p* < 0.05), consistent with the findings from GSE28551.

**Table 2.** GO and KEGG enrichment of 184 downregulated DEGs.

ONTOLOGY	ID	Description	GeneRatio	p.adjust	geneID	Zscore^a^
MF	GO:0001216	DNA-binding transcription activator activity	14/171	0.015	**ESRRG**/TP63/DLX3/ZNF750/NR6A1/FOXO4/GRHL1/ZFAT/TFAP2A/GCM1/TFCP2L1/GATA3/E2F2/TFAP2C	−3.742
MF	GO:0001228	DNA-binding transcription activator activity, RNA polymerase II-specific	14/171	0.015	**ESRRG**/TP63/DLX3/ZNF750/NR6A1/FOXO4/GRHL1/ZFAT/TFAP2A/GCM1/TFCP2L1/GATA3/E2F2/TFAP2C	−3.742
MF	GO:0001217	DNA-binding transcription repressor activity	11/171	0.018	ZIM2/OVOL1/PEG3/ZNF750/NFE2L3/IRX2/HIC2/TFAP2A/MSX2/GATA3/TFAP2C	−3.317
MF	GO:0001227	DNA-binding transcription repressor activity, RNA polymerase II-specific	11/171	0.018	ZIM2/OVOL1/PEG3/ZNF750/NFE2L3/IRX2/HIC2/TFAP2A/MSX2/GATA3/TFAP2C	−3.317
MF	GO:0008509	anion transmembrane transporter activity	10/171	0.035	SLC19A2/SLC27A6/SLC13A4/SLC22A11/SLC1A3/SLC13A3/SLC1A2/SLC38A9/SLC6A4/SLC52A1	−3.162
CC	GO:0005819	spindle	14/176	0.002	FAM83D/HMMR/RAB11FIP4/ASPM/CKAP2/KIF4A/BUB1B/CDCA8/CDC20/RACGAP1/CDK1/KIF18B/PRC1/AURKA	−3.742
CC	GO:0030496	midbody	9/176	0.008	RAB11FIP4/ASPM/ANLN/KIF4A/CDCA8/RACGAP1/CDK1/PRC1/AURKA	−3
BP	GO:0000280	nuclear division	16/170	0.006	TOP2A/ASPM/BMP7/ANLN/KIF4A/MSX2/BUB1B/CCNB2/CDCA8/CDC20/RACGAP1/KIF18B/IGF2/PRC1/CDCA5/AURKA	−4
BP	GO:0048285	organelle fission	16/170	0.006	TOP2A/ASPM/BMP7/ANLN/KIF4A/MSX2/BUB1B/CCNB2/CDCA8/CDC20/RACGAP1/KIF18B/IGF2/PRC1/CDCA5/AURKA	−4
BP	GO:0044772	mitotic cell cycle phase transition	15/170	0.006	TRIM71/SKP2/CDK6/FOXO4/CCNA2/ANLN/CDC25A/BUB1B/CCNB2/LZTS1/MELK/CDK1/EGFR/CDCA5/AURKA	−3.873
BP	GO:0048732	gland development	12/170	0.049	LAMA1/HGF/TP63/ATP7B/BMP7/MSX2/CCNB2/TFCP2L1/GATA3/EGFR/IGF2/AURKA	−3.464
BP	GO:0140014	mitotic nuclear division	12/170	0.006	BMP7/ANLN/KIF4A/BUB1B/CDCA8/CDC20/RACGAP1/KIF18B/IGF2/PRC1/CDCA5/AURKA	−3.464
BP	GO:0048608	reproductive structure development	12/170	0.049	LRP2/TP63/ZFP42/DLX3/ASPM/BMP7/STC2/GCM1/GATA3/EGFR/IGF2/TFAP2C	−3.464
BP	GO:0061458	reproductive system development	12/170	0.049	LRP2/TP63/ZFP42/DLX3/ASPM/BMP7/STC2/GCM1/GATA3/EGFR/IGF2/TFAP2C	−3.464
BP	GO:0045787	positive regulation of cell cycle	11/170	0.028	OVOL1/FAM83D/CDC25A/MSX2/SLC6A4/RACGAP1/CDK1/EGFR/IGF2/CDCA5/AURKA	−3.317
BP	GO:0051321	meiotic cell cycle	10/170	0.026	OVOL1/ZFP42/TOP2A/ASPM/CDC25A/MSX2/BUB1B/CCNB2/CDC20/AURKA	−3.162
BP	GO:0098813	nuclear chromosome segregation	10/170	0.032	FAM83D/TOP2A/KIF4A/BUB1B/CDCA8/CDC20/RACGAP1/KIF18B/PRC1/CDCA5	−3.162
BP	GO:1903046	meiotic cell cycle process	9/170	0.024	OVOL1/TOP2A/ASPM/CDC25A/MSX2/BUB1B/CCNB2/CDC20/AURKA	−3
BP	GO:0090068	positive regulation of cell cycle process	9/170	0.041	FAM83D/CDC25A/MSX2/RACGAP1/CDK1/EGFR/IGF2/CDCA5/AURKA	−3
BP	GO:0000819	sister chromatid segregation	9/170	0.024	TOP2A/KIF4A/BUB1B/CDCA8/CDC20/RACGAP1/KIF18B/PRC1/CDCA5	−3
KEGG	hsa04110	Cell cycle	10/82	0.000	SKP2/CDK6/CCNA2/CDC25A/BUB1B/CCNB2/CDC20/MCM2/CDK1/E2F2	−3.162

^a^ zscore: The larger the absolute value, the higher the gene may regulate the pathway.

### Downregulated ESRRG and abnormal mitochondria were found in ESA placental tissues

**Figure 2.**
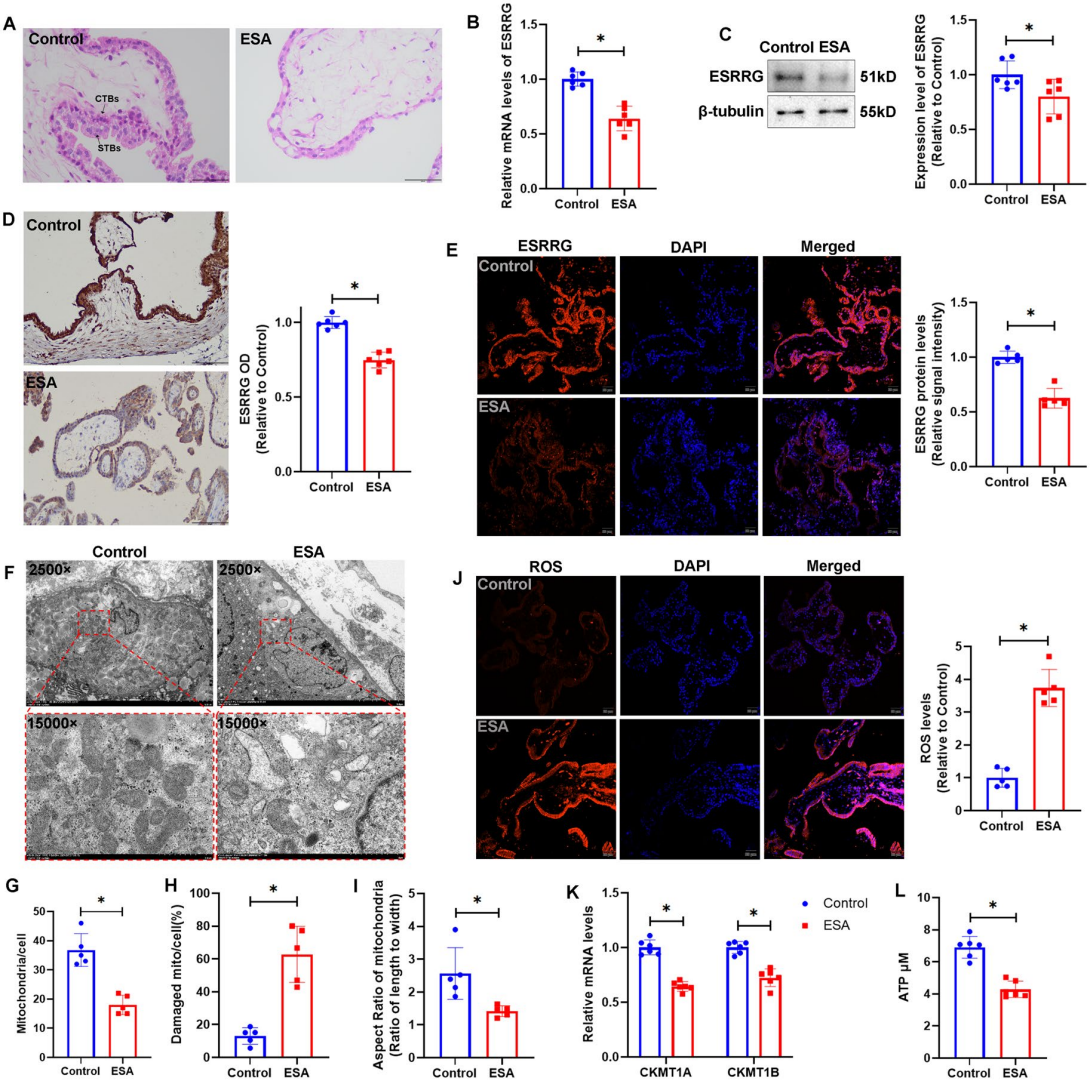
ESRRG expression levels and CTB mitochondrial conditions in human placental tissues. (A) HE-stained images of placental tissues from the control and ESA groups. Scale bar: 100 μm. (B) ESRRG mRNA levels were significantly lower in the ESA group than in the control group. The data are shown as the mean ± SD; *n* = 6 biological replicates. (C) Western blot analysis revealed that the ESRRG protein level was lower in the ESA group than in the control group. The data are shown as the mean ± SD; *n* = 6 biological replicates. (D) IHC images showing the expression of ESRRG in placental tissues from the control and ESA groups. Scale bar: 200 μm. The data are shown as the mean ± SD; *n* = 6 biological replicates. (E) IF images showing the expression of ESRRG in placental tissues from the control and ESA groups. Scale bar: 50 μm. The data are shown as the mean ± SD; *n* = 5 biological replicates. (F) Electron microscopy images of CTBs in placental tissues from the control and ESA groups. Representative images (out of 3 images obtained) are shown. Scale bar: 5 μm at 2500×; 1 μm at 15,000×. (G) Statistical analysis of the number of mitochondria per cell in the two groups *via* TEM (2,500×). The data are shown as the mean ± SD; *n* = 5 biological replicates. (H) The ratio of damaged mitochondria to the total number of mitochondria per cell. The data are shown as the mean ± SD; *n* = 5 biological replicates. (I) The aspect ratio of mitochondria per cell suggested that, in contrast to the predominantly narrow rod-shaped or elliptical mitochondria in control group trophoblasts, those in the ESA group trophoblasts showed a morphology dominated by plump elliptical or round shapes. (J) ROS levels in chorionic tissues were measured *via* DHE fluorescence. Scale bar: 50 μm. The data are shown as the mean ± SD; *n* = 5 biological replicates. (K) CKMT1A and CKMT1B mRNA levels were lower in the ESA group than in the control group. The data are shown as the mean ± SD; *n* = 6 biological replicates. (L) ATP levels were lower in the ESA group than in the control group. The data are shown as the mean ± SD; *n* = 6 biological replicates. (*p < 0.05).

In the villi tissues from the control group, the boundaries between syncytiotrophoblasts (STBs) and cytotrophoblasts (CTBs) were clearly defined. The cell morphology was relatively regular, the cell sizes were relatively uniform, and the cells were arranged in an orderly manner. STBs were in a multinucleated and fused state, covering the surface of the villi; CTBs were mononuclear and located beneath the STBs. In the villi tissues from the ESA group, the trophoblast layer was significantly thinner. The boundary between the STBs and CTBs was blurred, and the hierarchical structure was disordered. The trophoblasts varied in size and had irregular morphologies, with phenomena such as cell swelling, nuclear pyknosis, and nuclear fragmentation occurring (Figure 2A). ESRRG mRNA and protein levels decreased in the chorionic tissue of the ESA group (Figure 2B,C). IHC (Figure 2D) and IF (Figure 2E) results showed that ESRRG was expressed mainly in the trophoblast layer of villous tissues. The expression of ESRRG was significantly reduced in the ESA group. TEM (Figure 2F) revealed that in the control group, the mitochondria in the CTBs had an intact structure, with a smooth outer membrane, a matrix rich in granules, and relatively regularly-arranged cristae. In contrast, in the ESA group, the number of mitochondria was significantly reduced (Figure 2G), while the ratio of damaged mitochondria was higher (Figure 2H), and the mitochondria in the ESA group trophoblasts showed a morphology dominated by plump elliptical or round shapes (Figure 2I). The mitochondria were swollen, along with swelling of the inner membrane. The cristae were disordered, fused, and reduced in number. The number of granules in the matrix was significantly decreased, and vacuoles appeared. Furthermore, elevated levels of ROS were detected in the ESA cohort (Figure 2J). CKMT1A and CKMT1B mRNA levels decreased in the ESA group (Figure 2K), with a concomitant decrease in tissue ATP content (Figure 2L).

### ESRRG knockdown impaired the proliferation, invasion, and migration abilities and mitochondria impairment in HTR-8/Svneo cells

**Figure 3.**
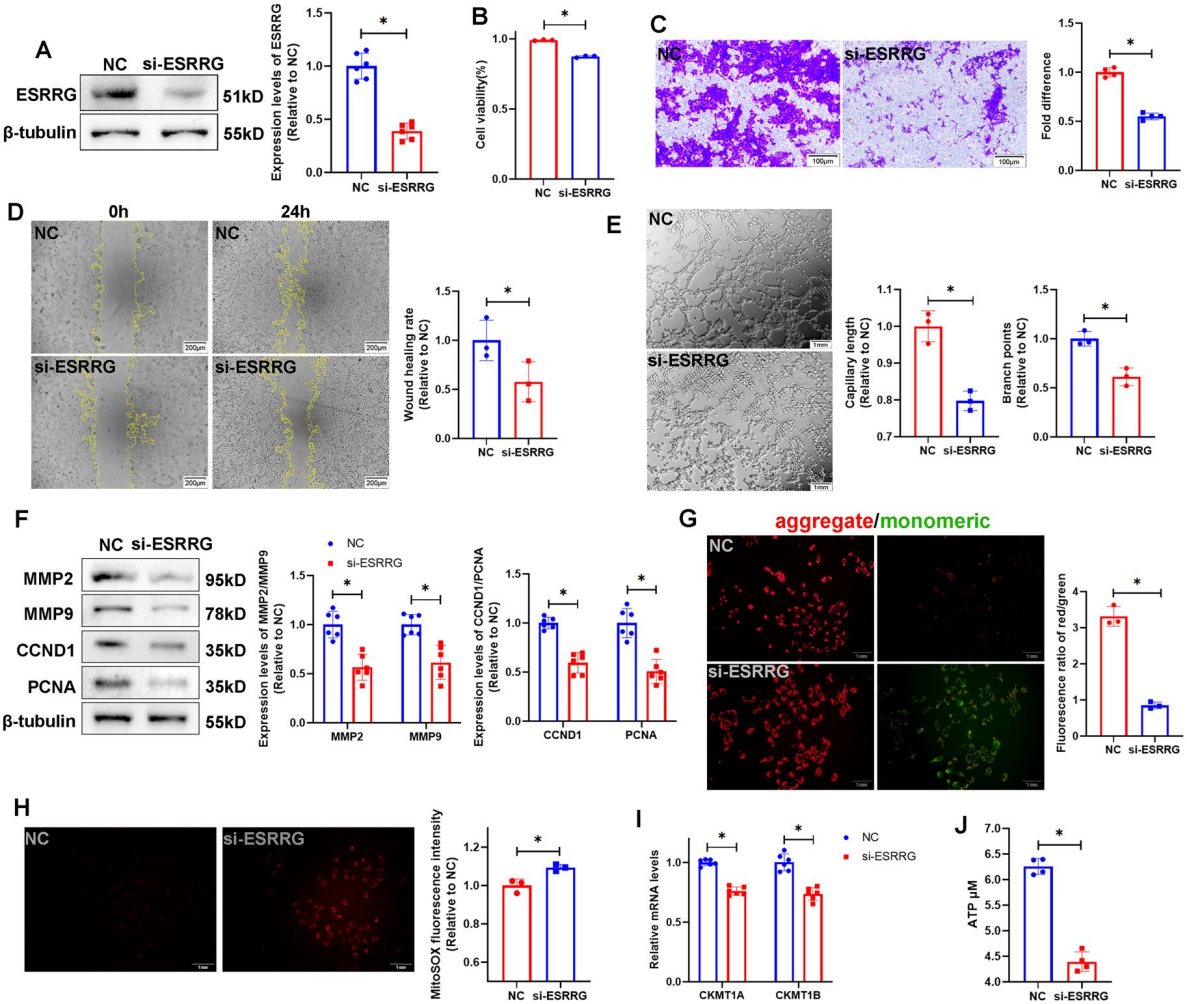
Effects of ESRRG knockdown on the functionality of NC and si-ESRRG-transfected HTR-8/SVneo cells. (A) Western blot analysis of ESRRG protein levels in NC and si-ESRRG-transfected cells. (B) A CCK-8 assay was used to measure cell proliferation. (C) Transwell assays were conducted to assess the invasion ability of the cells. The data are shown as the mean ± SD; *n* = 4 biological replicates. (D) A wound healing assay was used to test the migration ability of the cells. Images at 0 h and 24 h are presented. The data are shown as the mean ± SD; *n* = 3 biological replicates. (E) The tube-formation ability of the cells was assessed *via* a tube-formation assay. Images at 6 h are presented. The data are shown as the mean ± SD; *n* = 3 biological replicates. (F) Western blot detection of MMP2, MMP9, CCND1 and PCNA protein levels in cells. The data are shown as the mean ± SD; *n* = 6 biological replicates. (G) JC-1 assay was used to measure the MMP in HTR-8/SVneo cells, and representative images and quantitative analysis of red/green fluorescence intensity ratios were performed. The data are shown as the mean ± SD; *n* = 3 biological replicates. (H) MitoSOX fluorescence was used to assess the ROS levels in HTR-8/SVneo cells. The data are shown as the mean ± SD; *n* = 3 biological replicates. (I) CKMT1A and CKMT1B mRNA levels were lower in the si-ESRRG group than in NC. The data are shown as the mean ± SD; *n* = 6 biological replicates. (J) ATP levels were lower in the si-ESRRG group than in NC. The data are shown as the mean ± SD; *n* = 4 biological replicates. (*p < 0.05).

ESRRG was knocked down *via* siRNA transfection in HTR-8/Svneo cells (Figure 3A). The results of the CCK8 assay indicated decreased cell viability in the si-ESRRG group (Figure 3B). Transwell assays (Figure 3C) and cell migration assays (Figure 3D) revealed inhibition of invasion and migration capabilities in the si-ESRRG group. The tube-forming ability of the cells in the si-ESRRG group was significantly decreased (Figure 3E). Protein blotting revealed a reduction in the expression of the proliferation markers PCNA and CCND1 as well as the invasion markers MMP2 and MMP9 in the si-ESRRG group compared with those of the NC group (Figure 3F), suggesting that the downregulation of ESRRG adversely impacts the proliferation, invasion, and migration of HTR-8/Svneo cells. The MMP was significantly decreased in the si-ESRRG group (Figure 3G) and accompanied by an increase in the mROS level (Figure 3H), indicating that the downregulation of ESRRG may contribute to the impairment of mitochondria in trophoblasts, resulting in trophoblast dysfunction, which ultimately disrupts the functions of the placenta and may lead to foetal death. CKMT1A and CKMT1B mRNA levels decreased in the si-ESRRG group (Figure 3I), with a concomitant decrease in cell ATP content (Figure 3J), indicating that the decrease in ESRRG may impair mitochondrial energy production by reducing the expression of CKMT1A and CKMT1B.

### OE-Esrrg treatment increases embryo survival in an LPS-induced mouse miscarriage model

**Figure 4.**
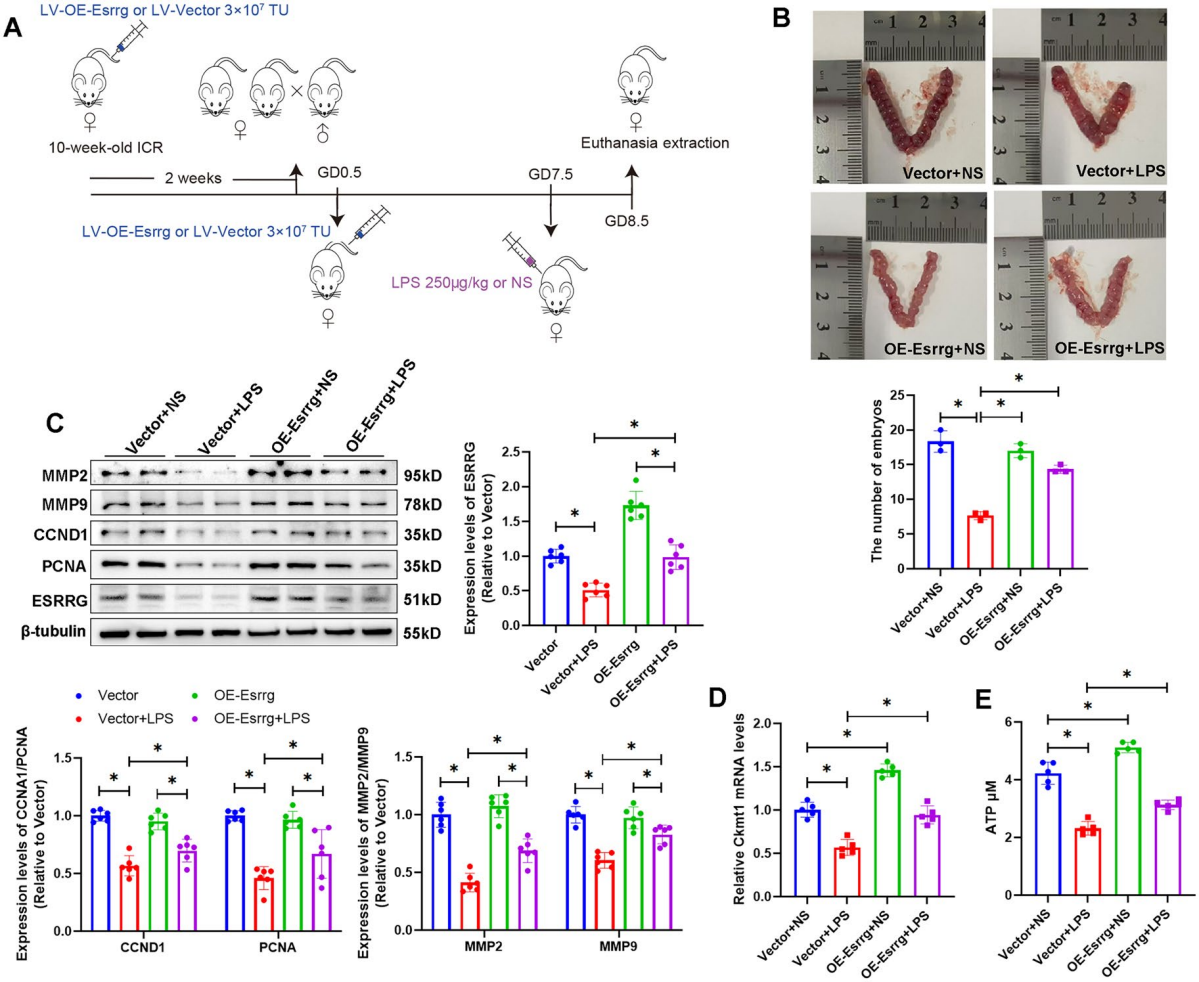
OE-Esrrg treatment alleviates ESAs induced by LPS in ICR mice. (A) Grouping and treatment methods of mice. *n* = 6 biological replicates. (B) Images and counts of viable embryos in the placentas of pregnant mice treated with OE-Esrrg or the vector. The data are shown as the mean ± SD; *n* = 3 biological replicates. (C) Western blot analyses of ESRRG, PCAN, CCND1, MMP2, and MMP9 in the placental tissues. The data are shown as the mean ± SD; *n* = 6 biological replicates. (D) Ckmt1 mRNA levels in the placental tissues. The data are shown as the mean ± SD; *n* = 5 biological replicates. (E) ATP levels in the placental tissues. The data are shown as the mean ± SD; *n* = 5 biological replicates. (*p < 0.05).

We injected lentiviral vectors *via* the tail vein to achieve global overexpression of Esrrg in ICR female mice. We then established an abortion model *via* induction with LPS to investigate whether the overexpression of Esrrg can reduce early abortion (Figure 4A). LPS successfully caused embryo loss in ICR mice during the early stage of pregnancy. Injection of OE-Esrrg significantly improved the survival rate of embryos in mice treated with LPS (Figure 4B). Western blot analysis (Figure 4C) revealed that ESRRG levels were lower in the Vector + LPS group than in the Vector group, establishing a correlation between the downregulation of ESRRG and LPS-induced ESA. LV-OE-Esrrg increased the expression levels of MMP2, MMP9, CCND1 and PCNA in the LPS-induced miscarriage mice, indicating that it ameliorated the functionality of trophoblast cells. Ckmt1 mRNA levels were significantly reduced in the placental tissues from LPS-induced miscarriage group (Figure 3D), which correlated with diminished tissue ATP content (Figure 3E). In contrast, OE-Esrrg effectively reversed these changes, upregulating Ckmt1 expression and restoring ATP levels. These results suggested that OE-Esrrg treatment could protect against ESAs induced by LPS in mice by improving the impaired function of trophoblasts.

## Discussion

ESA is a prevalent complication during pregnancy. To investigate the molecular mechanisms associated with this condition, we analysed the dataset GSE123719 and focused on one of the downregulated genes, ESRRG. ESRRG is expressed in the trophoblast, and its expression level in the placenta gradually increases with the progression of gestational weeks, suggesting that it may play an important role in maintaining normal pregnancy. Chorionic tissues from ESA patients and normal controls were collected, and a decrease in ESRRG mRNA and protein expression was detected in ESAs, accompanied by abnormal morphology of mitochondria in CTBs. ESRRG knockdown led to reduced expression of markers associated with trophoblast cell proliferation and invasion as well as mitochondrial function. Esrrg overexpression in an LPS-induced abortion mouse model increased the number of remaining embryos and improved the protein levels of cellular function and mitochondrial function markers in trophoblast cells. These results indicate that ESRRG serves a protective role in embryos during early gestation by supporting the proliferation, migration and invasion functions of trophoblast cells through the regulation of mitochondrial function.

The proliferation, invasion and migration abilities of trophoblast cells are crucial for embryo implantation, placental development and the maintenance of a normal pregnancy, and these processes require a large amount of energy. Mitochondria are the core of metabolic activities in cells and tissues and are responsible for meeting the metabolic and energy requirements to maintain the normal functions of trophoblast cells and the continuous growth of the placenta [23,29,30]. Mitochondria provide a large amount of energy for trophoblast cells through the oxidative phosphorylation and glycolysis pathways and support the gradually increasing material transport function of trophoblasts to meet the growth needs of the embryo [31]. Research has shown that mitochondrial dysfunction can lead to decreases in the proliferation, invasion, and migration functions of trophoblast cells, which are closely associated with spontaneous abortion [32–34]. For example, decreased expression of cytochrome oxidase subunit 4 isoform 1 (COX4I1) results in mitochondrial dysfunction, subsequently impairing the proliferation, invasion, and migration of trophoblast cells, thereby increasing the likelihood of spontaneous abortion [35]. Moreover, before 12 weeks of gestation, the maternal–foetal circulation remains incompletely developed. In this context, a hypoxic environment facilitates the antiapoptotic capacity and rapid proliferation of CTBs [36]. During the initial phase of maternal–foetal circulation establishment, which occurs between 10 and 12 weeks of gestation, increased blood perfusion leads to an increase in local ROS within the trophoblast. This increase is essential for maintaining the normal invasive ability of STBs, promoting angiogenesis, and facilitating placental remodelling [37]. Conversely, an untimely increase in ROS (between 8 and 9 weeks of gestation) can induce apoptosis of CTBs, degeneration of STBs, and impairment of their invasive function. These adverse effects can subsequently compromise placental development and may even result in early spontaneous abortion. Mitochondrial dysfunction generates excessive ROS, which in turn exacerbates existing mitochondrial dysfunction, thus establishing a vicious cycle [38–40]. ROS-induced endoplasmic reticulum (ER) stress can also lead to apoptosis of trophoblast cells and decidual cells, resulting in early pregnancy loss [41,42]. Through observation under *via* TEM, we found obvious morphological changes in the mitochondria of cytotrophoblasts in patients with ESAs. Fluorescent probe detection revealed a significant increase in ROS in the chorionic tissues of ESA patients. These findings suggest that mitochondrial dysfunction is closely associated with the occurrence of spontaneous abortion, which is consistent with the results of previous studies.

ESRRG functions as a transcriptional regulator that modulates the expression of various genes associated with mitochondrial biogenesis, lipid metabolism, and glycolysis [43]. For example, in brown adipose tissue, ESRRG maintains the metabolic response to adrenergic signalling and thermogenic function by regulating mitochondrial biogenesis and oxidative capacity [44]. ESRRG interacts with peroxisome proliferator-activated receptor gamma coactivator 1 alpha (PPARGC1A) to maintain the energy metabolism homeostasis of the heart by regulating mitochondrial functions and the expression of metabolism-related genes [45]. Knockout of ESRRG impairs mitochondrial functions, which in turn impairs spatial learning and memory abilities in the hippocampus [46]. It also leads to a decrease in the expression of mitochondrial genes and the number of mitochondria in the dopaminergic neurons of mice, thereby triggering pathological features similar to Parkinson’s disease [47]. ESRRG deficiency leads to mitochondrial dysfunction in pancreatic acinar cells, which in turn triggers autophagic dysfunction, ER stress, and increased ROS, ultimately results in diseases such as pancreatitis and pancreatic cancer [48]. The selective ESRRG agonist DY131 has been shown to increase the expression of Esrrg by activating its transcriptional activity and improving mitochondrial function to alleviate acute kidney injury in mice [49]. These findings suggest that ESRRG is capable of engaging in the modulation of diverse pathophysiological processes through the regulation of mitochondrial function. In our study, ESRRG knockdown resulted in mitochondrial dysfunction and decreased the proliferation, migration, invasion and tube formation abilities of HTR-8/SVneo cells, indicating that ESRRG downregulation can lead to trophoblast dysfunction by impairing both mitochondrial structure and function. Among the potential mechanisms at play, ESRRG may modulate trophoblast energy metabolism by influencing the expression of genes associated with mitochondrial biogenesis and oxidative metabolism. This regulatory role is crucial for the differentiation of trophoblast cells, which are vital for the appropriate development and functionality of the placenta [23]. The vascular regulatory function of trophoblasts is crucial for placental development. Trophoblasts secrete various vasoactive factors and related receptors. HTR-8/SVeno cells express Vascular Endothelial Growth Factor-A (VEGF-A) to form tubular network structures on Matrigel through cell-cell and cell-matrix interactions [50]. Trophoblasts express VEGF-A, which enhances tubulogenesis of themselves and adjacent endothelial cells *via* autocrine and paracrine effects to induce vascular sprouting. They also express platelet-derived growth factor (Pdgf), Angiopoietin-1 (Ang-1), and the Tie2 receptor to stabilise new blood vessels. Interacting with endothelial cells and pericytes, trophoblasts participate in constructing the vascular network in placental villous spaces to ensure maternal-fetal material exchange [51,52]. In early pregnancy, extravillous trophoblasts secrete matrix metalloproteinases to degrade extracellular matrix, invading uterine spiral arteries to promote remodelling of placental vascular networks, transforming them from high-resistance, low-flow vessels into low-resistance, high-flow ones [53]. The synthetic and secretory functions of trophoblasts require substantial energy. Knocking down ESRRG weakened the tubulogenic capacity of HTR-8/SVeno cells, possibly due to decreased ATP from impaired mitochondrial function, leading to reduced secretory function. Furthermore, a reduction in the expression levels of ESRRG and its downstream target genes, such as HSD11B2 and CYP19A1, in trophoblast cells may lead to decreased invasiveness and increased apoptosis rates. This occurrence is associated with a decrease in mitochondrial abundance and subsequent mitochondrial dysfunction, which ultimately contributes to placental dysfunction [54,55]. Our bioinformatics analysis and experimental results showed that the expression levels of two genes, CKMT1A and CKMT1B, which are involved in the regulation of mitochondrial function, were significantly decreased in the ESA group. Moreover, through a search of the results of the ChIP-seq dataset GSE104905, we found that ESRRG might be enriched in the promoter-transcription start site (TSS) region of the CKMT1 gene. These findings suggest that ESRRG may regulate genes related to the mitochondrial function of chorionic tissues by modulating the expression of CKMT1B and CKMT1A.

We further elucidated the role of Esrrg in trophoblast function and embryonic development *in vivo*. In the LPS-induced abortion mouse model, the overexpression of Esrrg increased embryo survival rates and upregulated the expression of markers associated with proliferation and invasion abilities. This phenomenon may involve multiple mechanisms, as follows: to some extent, the upregulated Esrrg improved the damaged downstream signalling pathways; partially restored the expression of mitochondrial-related genes; and enhanced mitochondrial function; thereby partially restoring the energy supply of trophoblast cells. The upregulated Esrrg can reduce the production of ROS by improving mitochondrial function and decrease the damage caused by ROS to the functionality of trophoblast cells as well as its pro-apoptotic effect by increasing the antioxidant capacity of cells. Moreover, it can mitigate the adverse effects of excessive ROS on embryonic development [53]. In the placentas of mice with Esrrg knockdown, the expression of VEGFA increased significantly, leading to abnormal placental angiogenesis and placental dysfunction. These findings suggest that ESRRG can also promote the normal formation of placental blood vessels by regulating the expression of vascular endothelial growth Factor A (VEGFA), thus maintaining a normal pregnancy [56]. Esrra, in collaboration with PPARGC1A, plays a crucial role in regulating uterine decidualization during the early stages of pregnancy. This regulatory function is instrumental in facilitating the implantation and subsequent development of fertilized eggs [23,57]. Given that Esrrg has a high degree of homology with Esrra, possesses similar structural and functional properties, and is also capable of interacting synergistically with PPARGC1A, Esrrg may be involved in the regulatory processes governing the endometrium and the intrauterine microenvironment. In summary, ESRRG is likely to improve the prognosis of mice prone to abortion through multiple mechanisms, including the amelioration of mitochondrial function and the modulation of oxidative stress.

## Conclusions

Our study confirmed for the first time that the downregulation of ESRRG may play a crucial role in the occurrence and development of ESAs by affecting the development and function of the placenta through the regulation of mitochondrial function and energy metabolism of trophoblast cells, suggesting that an ESRRG-targeted strategy has great potential in the clinical treatment of ESAs. There are several limitations to our study. The sample size of human/mouse subjects is relatively small, and the regulatory mechanisms upstream of ESRRG in ESA, as well as the molecular mechanisms by which ESRRG regulates mitochondrial function, remain to be further explored. The potential effects of ESRRG downregulation should be validated and explored not only in the LPS-induced miscarriage mouse model but also in other miscarriage models. These aspects will be investigated in our future research.

## Supplementary Material

Fig4C PCNA with group and marker messages.Tif

Fig4C CCND1 with group and marker messages.Tif

Fig4C BetaTubulin with group and marker messages.Tif

Fig2C ESRRG with group and marker messages.tif

Fig3F MMP2 with group and marker messages.Tif

Fig3F BetaTubulin with group and marker messages.Tif

Fig4C MMP2 with group and marker messages.Tif

Fig3F PCNA with group and marker messages.Tif

Fig3F CCND1 with group and marker messages.Tif

Revised The ARRIVE guidelines author checklist.pdf

Fig3A ESRRG with group and marker messages.Tif

Clean copy of Revised Manuscript.docx

Table S1.docx

corrected Fig2C BetaTubulin with group and marker messages.tif

Fig4C ESRRG with group and marker messages.Tif

Fig4C MMP9 with group and marker messages.Tif

Fig3A BetaTubulin with group and marker messages.Tif

Fig3F MMP9 with group and marker messages.Tif

## Data Availability

The data that support the findings of this study are available from the corresponding author, Sha Lv, upon reasonable request.
