# Supplementary figures and images for: ESRRG downregulation in early spontaneous abortion induces mitochondrial damage, leading to impaired trophoblast function
Source: Ann Med. 2026 Feb 2;58(1):2622749. doi: 10.1080/07853890.2026.2622749 (PMC12865851; doi:10.1080/07853890.2026.2622749)

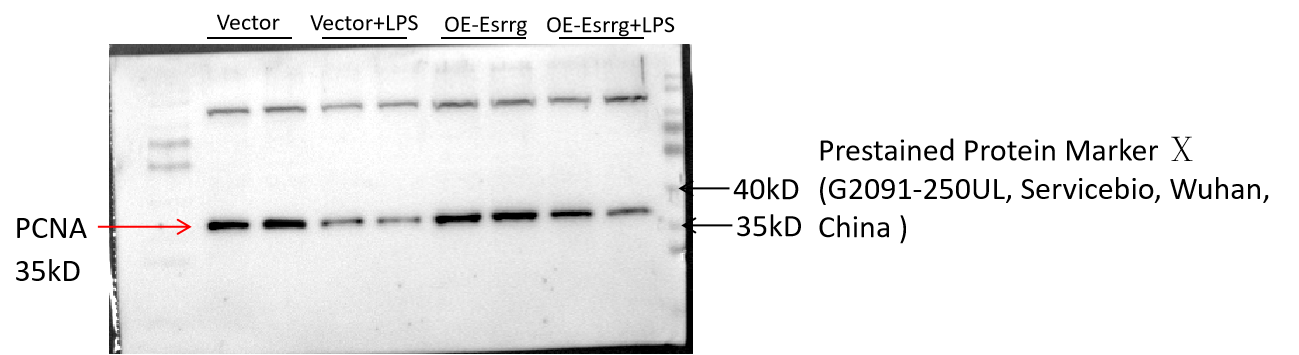

Supplement: Fig4C PCNA with group and marker messages.Tif [file IANN_A_2622749_SM1321.tif]

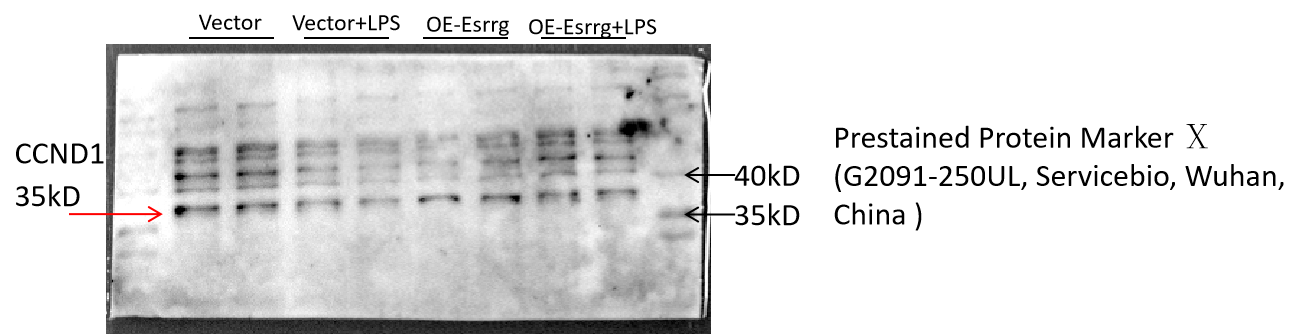

Supplement: Fig4C CCND1 with group and marker messages.Tif [file IANN_A_2622749_SM1320.tif]

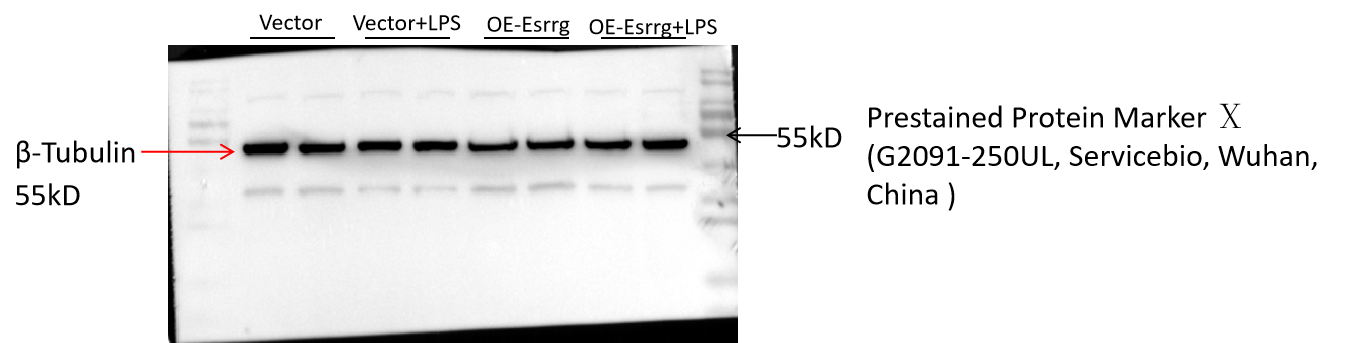

Supplement: Fig4C BetaTubulin with group and marker messages.Tif [file IANN_A_2622749_SM1319.tif]

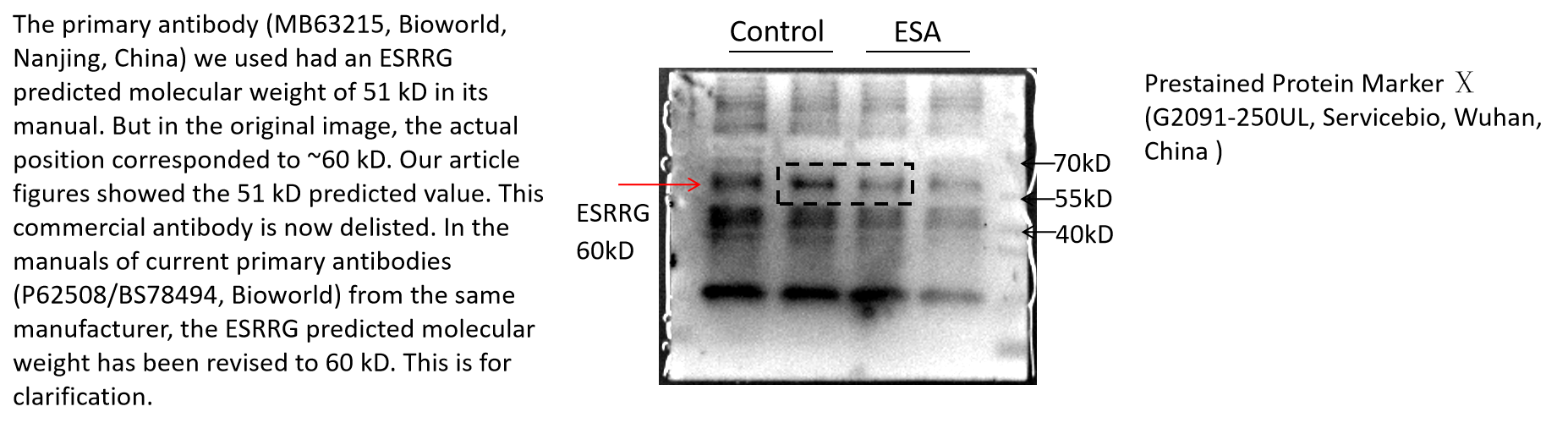

Supplement: Fig2C ESRRG with group and marker messages.tif [file IANN_A_2622749_SM1318.tif]

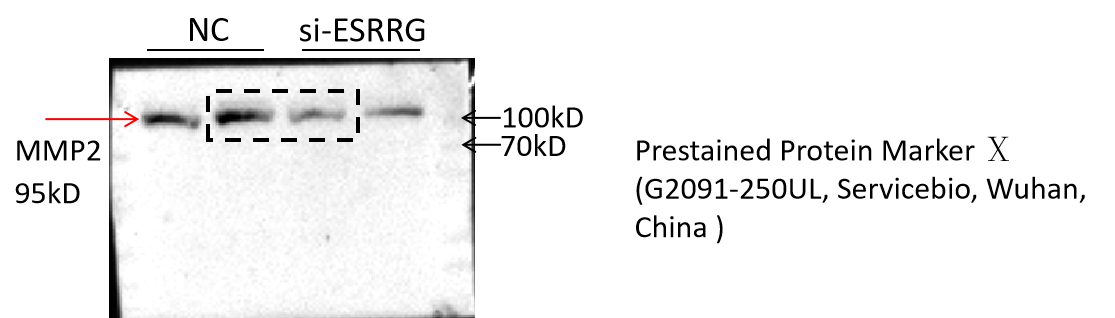

Supplement: Fig3F MMP2 with group and marker messages.Tif [file IANN_A_2622749_SM1317.tif]

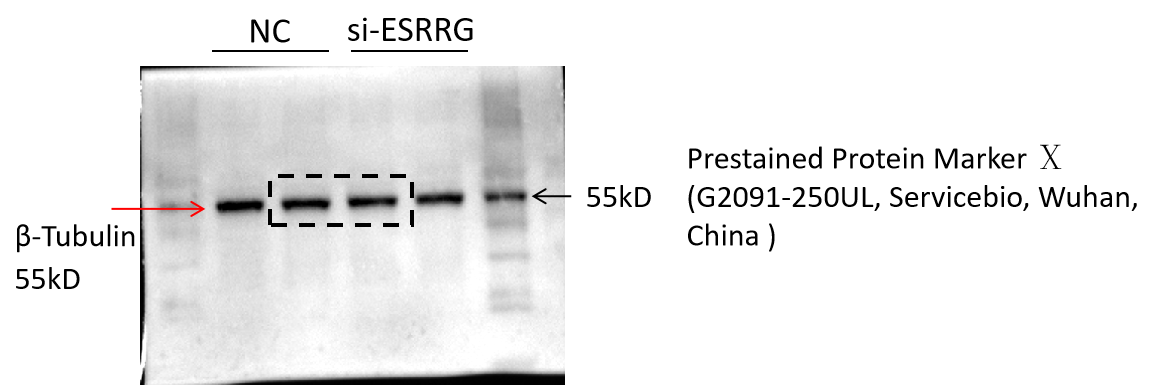

Supplement: Fig3F BetaTubulin with group and marker messages.Tif [file IANN_A_2622749_SM1316.tif]

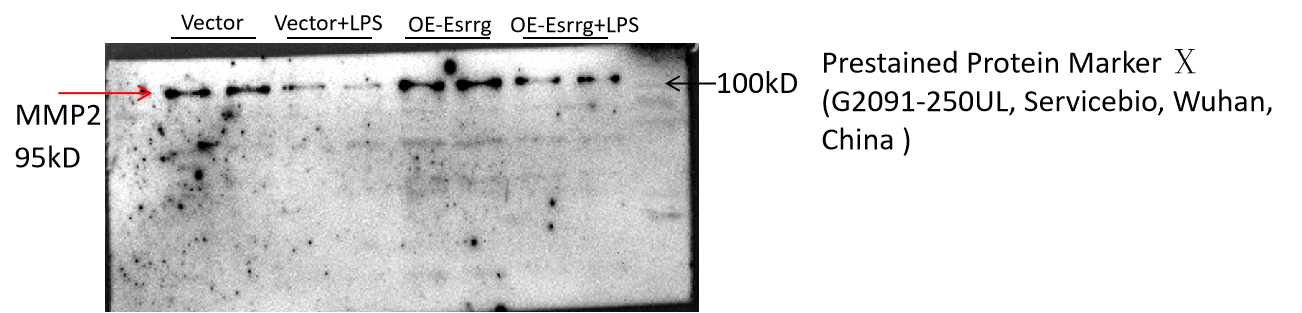

Supplement: Fig4C MMP2 with group and marker messages.Tif [file IANN_A_2622749_SM1315.tif]

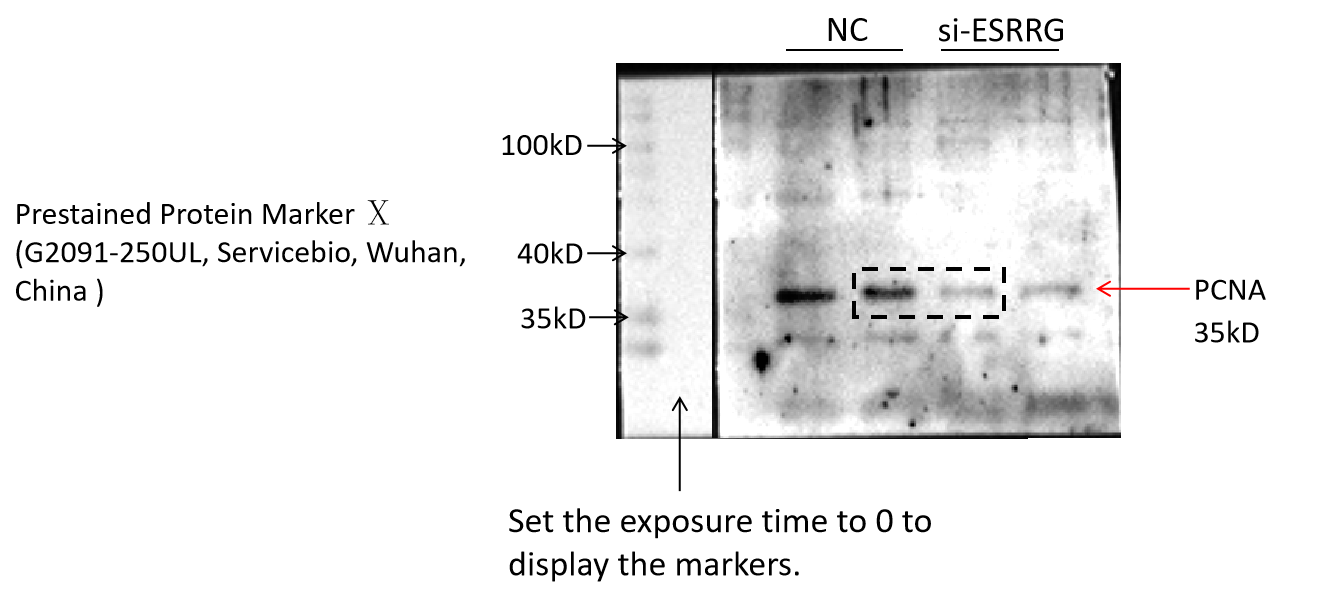

Supplement: Fig3F PCNA with group and marker messages.Tif [file IANN_A_2622749_SM1314.tif]

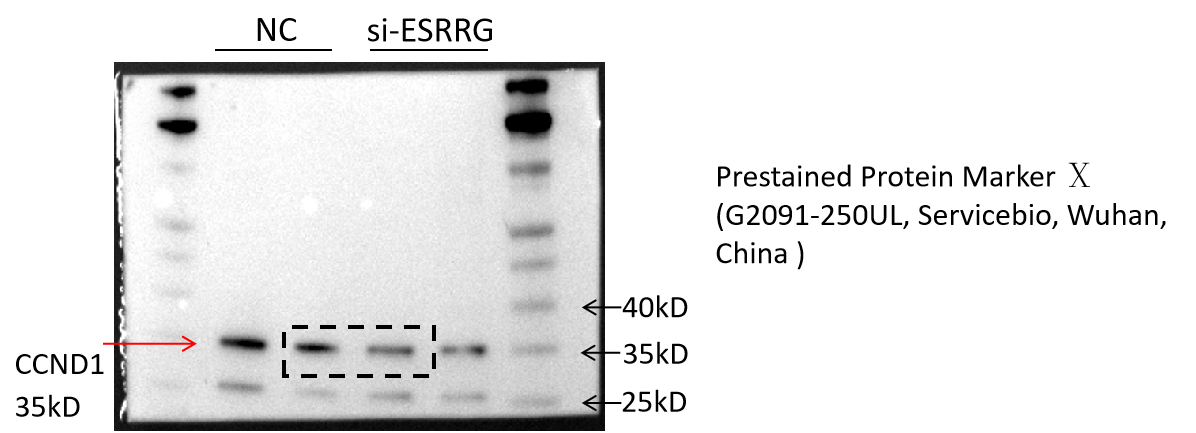

Supplement: Fig3F CCND1 with group and marker messages.Tif [file IANN_A_2622749_SM1313.tif]

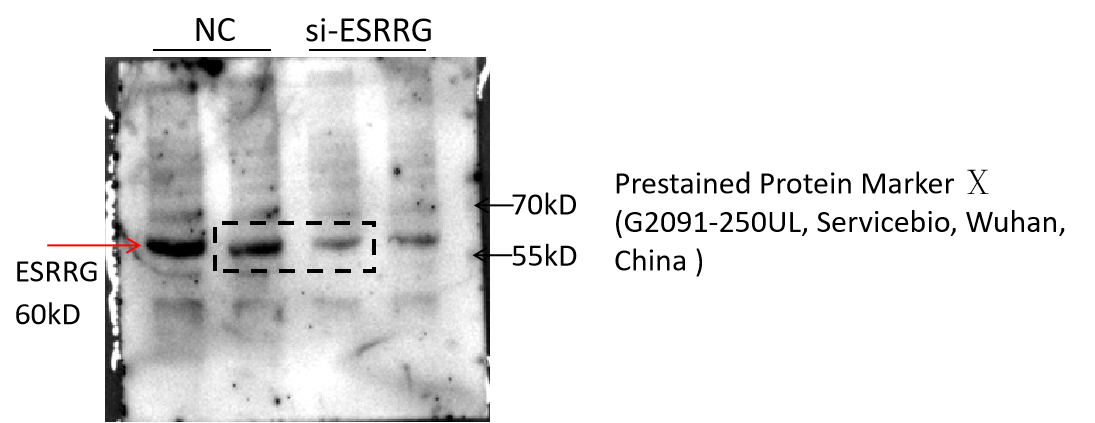

Supplement: Fig3A ESRRG with group and marker messages.Tif [file IANN_A_2622749_SM1311.tif]

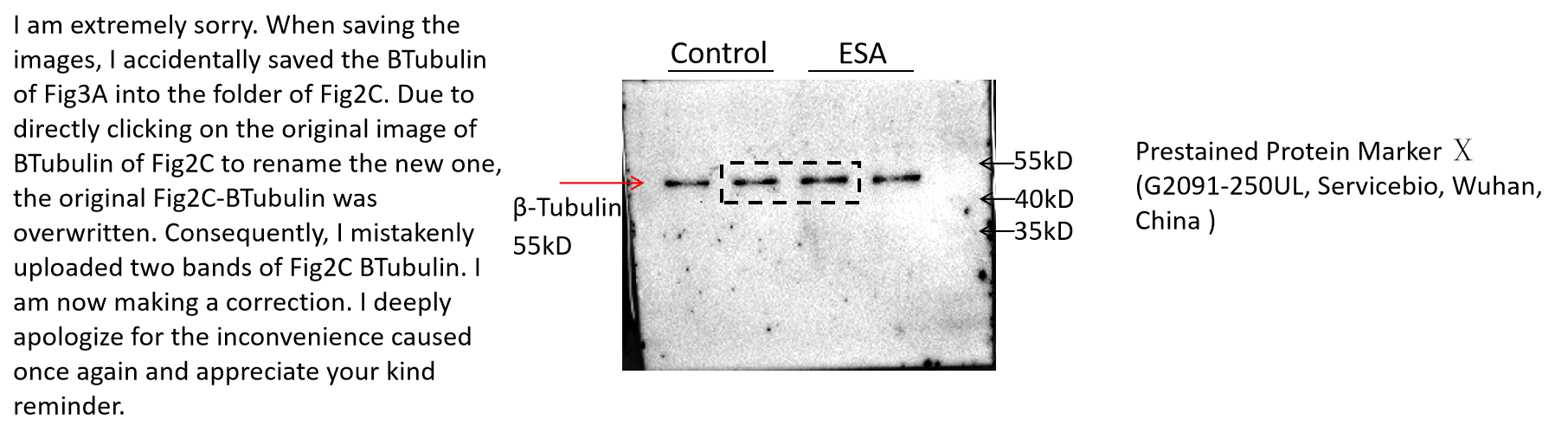

Supplement: corrected Fig2C BetaTubulin with group and marker messages.tif [file IANN_A_2622749_SM1308.tif]

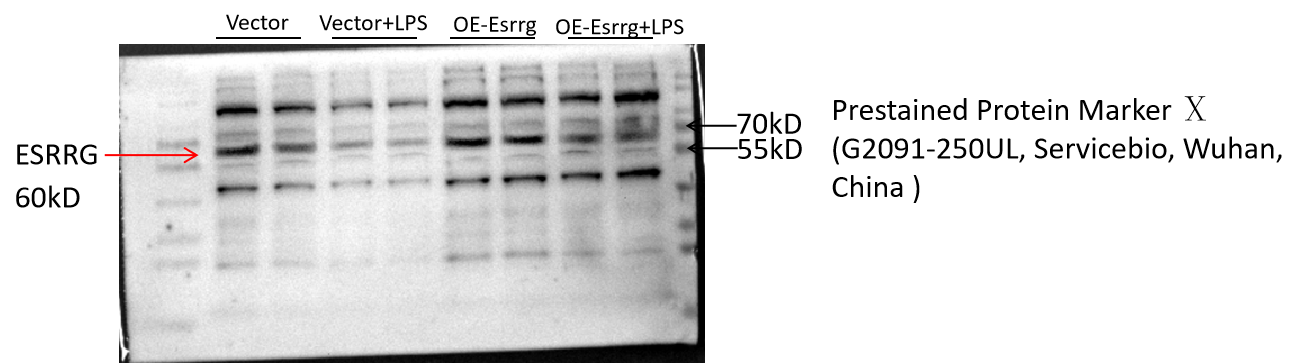

Supplement: Fig4C ESRRG with group and marker messages.Tif [file IANN_A_2622749_SM1307.tif]

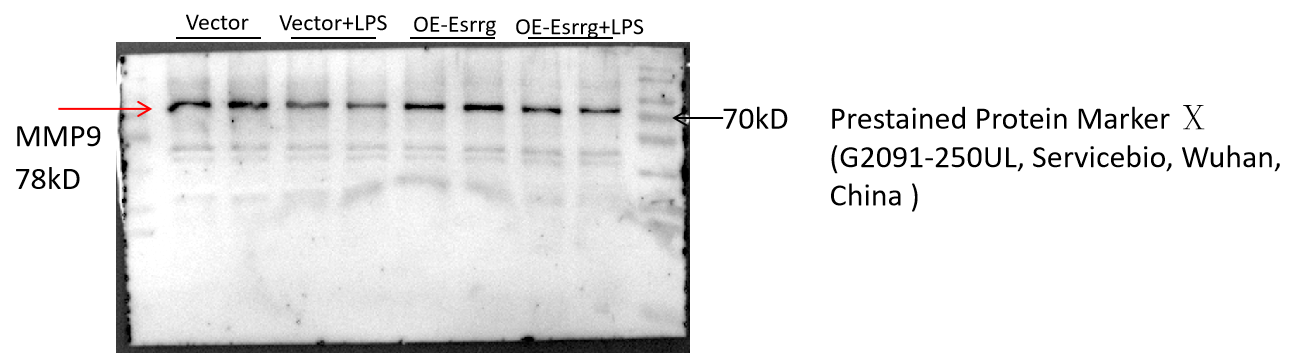

Supplement: Fig4C MMP9 with group and marker messages.Tif [file IANN_A_2622749_SM1306.tif]

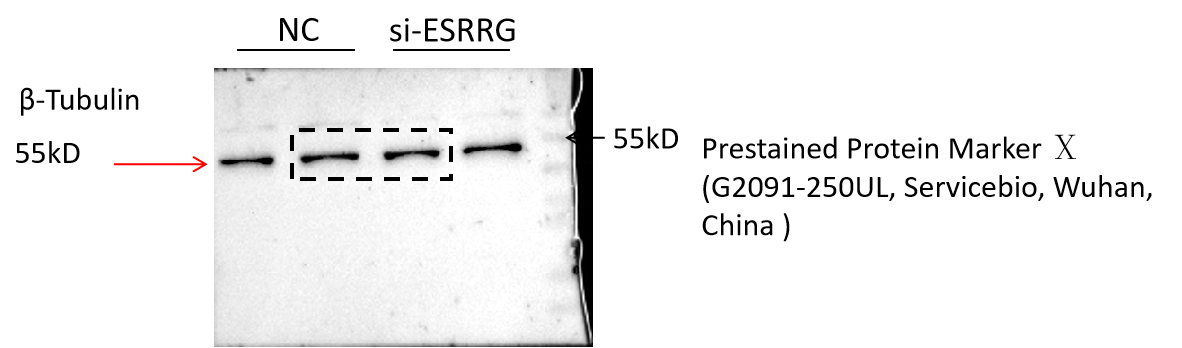

Supplement: Fig3A BetaTubulin with group and marker messages.Tif [file IANN_A_2622749_SM1305.tif]

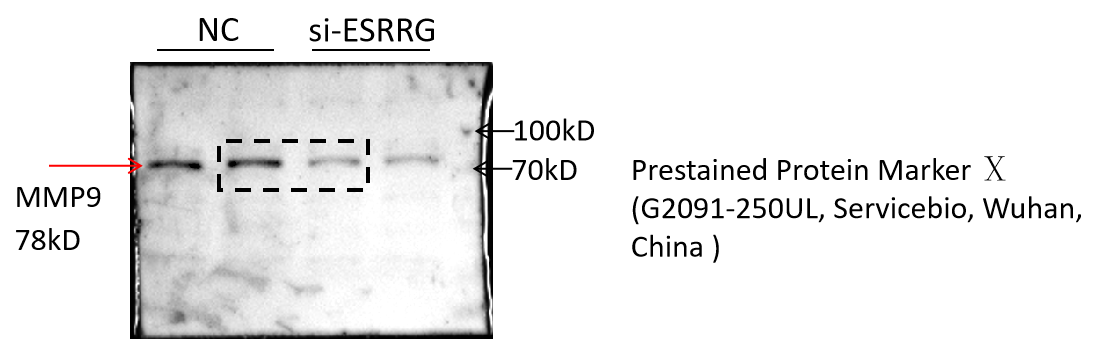

Supplement: Fig3F MMP9 with group and marker messages.Tif [file IANN_A_2622749_SM1304.tif]
