# Supplementary material for: ESRRG downregulation in early spontaneous abortion induces mitochondrial damage, leading to impaired trophoblast function
Source: Ann Med. 2026 Feb 2;58(1):2622749. doi: 10.1080/07853890.2026.2622749 (PMC12865851; doi:10.1080/07853890.2026.2622749)
Supplement: Table S1.docx [file IANN_A_2622749_SM1309.docx]

Table S1. Primary and secondary antibodies used for western blot (WB), Immunofluorescence (IF) or Immunohistochemistry (IHC) analysis.

| MARKER | DILUTION | DISTRIBUTOR/SOURCE  (CATALOG NUMBER) |
| --- | --- | --- |
| Rabbit monoclonal IgG to β-Tubulin | WB 1:5000 | ABclonal (A12289) |
| Mouse monoclonal IgG to ESRRG | WB 1:2000  IF 1:200  IHC 1:200 | Bioworld Biotech (MB63215) |
| Rabbit Polyclonal IgG to MMP2 | WB 1:1000 | Immunoway (YT2798) |
| Rabbit Polyclonal IgG to MMP9 | WB 1:1000 | Immunoway (YT1892) |
| Rabbit Polyclonal IgG to CCND1 | WB 1:1000 | Solarbio (K007413P) |
| Rabbit Polyclonal IgG to PCNA | WB 1:1000 | Wanleibio (WL03213) |
| Goat Anti Rabbit IgG (H+L) (HRP) | WB 1:10000 | Immunoway (RS0002) |
| Goat Anti Mouse IgG (H+L) (HRP) | WB 1:10000 | Immunoway (RS0001) |
| Enzyme-labeled goat anti-mouse/rabbit IgG polymer | IHC 1:1 | ZSGB-BIO (PV9000) |
| CoraLite594 – conjugated Goat Anti-Rabbit IgG (H+L) | IF 1:300 | Proteintech (SA00013-4) |
